# A 3-slice cardiac quantitative native and post-contrast T1 and T2 MRI protocol requiring only four BHs using a 72-channel receive array coil

**DOI:** 10.3389/fcvm.2023.1285206

**Published:** 2023-11-27

**Authors:** Hugo Klarenberg, Mark Gosselink, Fasiha Siddiqui, Bram F. Coolen, Aart J. Nederveen, Tim Leiner, Hildo J. Lamb, S. Matthijs Boekholdt, Gustav J. Strijkers, Martijn Froeling

**Affiliations:** ^1^ Department of Biomedical Engineering and Physics, Amsterdam Cardiovascular Sciences, Amsterdam University Medical Centers, University of Amsterdam, Amsterdam, Netherlands; ^2^ Department of Radiology, University Medical Center Utrecht, Utrecht, Netherlands; ^3^ Department of Physiology, Amsterdam Cardiovascular Sciences, Amsterdam University Medical Centers, Vrije Universiteit Amsterdam, Amsterdam, Netherlands; ^4^ Department of Radiology and Nuclear Medicine, Amsterdam University Medical Centers, University of Amsterdam, Amsterdam, Netherlands; ^5^ Department of Radiology, Leiden University Medical Center, Leiden, Netherlands; ^6^ Department of Cardiology, Amsterdam Cardiovascular Sciences, Amsterdam University Medical Centers, University of Amsterdam, Amsterdam, Netherlands

**Keywords:** T1 mapping, T2 mapping, extracellular volume, quantitative magnetic resonance imaging, multi-slice imaging, parallel imaging, under sampling, coil development

## Abstract

**Introduction:**

Current practice to obtain left ventricular (LV) native and post-contrast T1 and T2 comprises single-slice readouts with multiple breath-holds (BHs). We propose a multi-slice parallel-imaging approach with a 72-channel receive-array to reduce BHs and demonstrate this in healthy subjects and hypertrophic cardiomyopathy (HCM) patients.

**Methods:**

A T1/T2 phantom was scanned at 3 T using a 16-channel and a novel 72-channel coil to assess the impact of different coils and acceleration factors on relaxation times. 16–18 healthy participants (8 female, age 28.4 ± 5.1 years) and 3 HCM patients (3 male, age 55.3 ± 4.2 years) underwent cardiac-MRI with the 72-channel coil, using a Modified Look-Locker scan with a shared inversion pulse across 3 slices and a Gradient-Spin-Echo scan. Acceleration was done by sensitivity encoding (SENSE) with accelerations 2, 4, and 6. LV T1 and T2 values were analyzed globally, per slice, and in 16 segments, with SENSE = 2 as the reference.

**Results:**

The phantom scans revealed no bias between coils and acceleration factors for T1 or T2, except for T2 with SENSE = 2, which resulted in a bias of 8.0 ± 6.7 ms (*p* < 0.001) between coils. SENSE = 4 and 6 enabled T1 mapping of three slices in a single BH, and T2 mapping of three slices within two BHs. In healthy subjects, T1 and T2 values varied. We found an average overestimation of T1 in 3 slices of 25 ± 87 ms for SENSE = 4 and 30 ± 103 ms using SENSE = 6, as compared to SENSE = 2. Acceleration resulted in decreased signal-to-noise; however, visually insignificant and without increased incidence of SENSE-artifacts. T2 was overestimated by 2.1 ± 5.0 ms for SENSE = 4 and 6.4 ± 9.7 ms using SENSE = 6, as compared to SENSE = 2. Native and post-contrast T1 measurements with SENSE = 4 and ECV quantification in HCM patients was successful.

**Conclusion:**

The 72-channel receiver-array coil with SENSE = 4 and 6, enabled LV-tissue characterization in three slices. Pre- and post-contrast T1 maps were obtained in a single BH, while T2 required two BHs.

## Introduction

Quantitative Magnetic Resonance Imaging (qMRI) via native T2 and native and post-contrast T1 mapping is increasingly used in cardiac imaging to guide clinical care (1). Native and post-contrast T1 mapping allows for extracellular volume (ECV) estimation (2). It has been shown that native T1 times and ECVs increase in cardiac pathologies such as dilated (DCM) and hypertrophic cardiomyopathies (HCM). Conversely, certain cardiac pathologies, such as Anderson-Fabry disease, lead to decreased native T1 values. T2 mapping is regularly used to quantify the presence of myocardial edema or inflammation. Recently native T1 and T2 mapping have also been used to assess myocardial perfusion which can be of use in patients suspected of having coronary artery disease (CAD) (3, 4). Clinically, T1 and T2 maps are typically acquired at 3 different short axis (SAx) slice locations at base, mid-ventricular, and apical levels. A thorough examination of the entire heart is obtained through this comprehensive analysis (5). The downside of this approach is that each acquired slice requires 1 breath-hold (BH) of 11–15 s. Thus, a full native T1 and T2 mapping examination takes at least 6 BHs and several minutes, which increases to 9 BHs if post-contrast T1 maps are included. Many cardiac patients struggle with breath-holding, making it challenging for them. Additionally, respiratory motion artifacts are frequently observed if BHs are inconsistent or incomplete. Hence, there is a compelling need to reduce the number of BHs, which can be accomplished by accelerating the image acquisition process.

Cardiac T1 maps are commonly acquired using a Modified Look Locker sequence (MOLLI) (6). An often-used pattern for native T1 quantification is 5(3)3, indicating signal acquisition after a first inversion pulse during 5 heartbeats, followed by a 3 heartbeats recovery, and a second inversion pulse with 3 heartbeats readout. For some vendors, the numbers in the pattern refer to seconds instead of heartbeats, indicated by 5s(3s)3s. For T1 post-contrast, the pattern is adapted to increase the number of readouts with short inversion times since T1 is lower, for example to 4(1)3(1)2. Both patterns are designed to fit within a single BH. T2 maps can be obtained using a T2-preparation module or Gradient-Spin-Echo (GraSE) sequence, requiring multiple BHs to acquire multiple echo times and obtain sufficient heart coverage (7).

In recent literature, various methods have been introduced to address the challenges posed by lengthy scan times and the need for many BHs. These approaches involve the development of new multi-parametric mapping approaches, multi-band imaging techniques, sparse sampling schemes, and model-based reconstruction methods (8–11). Most of the approaches enable free-breathing and/or ultra-short breath-holds. Despite this progress, the practical implementation of myocardial T1 and T2 mapping in routine clinical settings has encountered challenges and complexities. As of now, achieving a widespread and seamless integration of these methods into clinical practice has not been a straightforward task (12, 13). Hence, the practical utilization of proposed acceleration solutions in clinical settings is not currently accessible due to several factors. One such factor is the complexity and extended duration required for offline reconstruction, preventing instant evaluation of scan quality, success, or failure. This limitation impedes the immediate assessment of the scans and contributes to the unavailability of these accelerating solutions in routine clinical practice.

In this paper, we have taken a pragmatic solution for speeding up native and post-contrast T1 as well as T2 mapping acquisitions, by exploiting a recently developed 72-channel receive array coil that enables high parallel imaging acceleration (14–19). This has the distinct advantage that one can utilize the vendor-supplied parallel imaging reconstruction algorithms, such as sensitivity encoding (SENSE), without the need for off-line reconstruction making the T1 and T2 maps directly available to the MRI operator for evaluation.

The objective of this research was to examine the efficacy of a 72-channel receive array coil in acquiring multiple slices of native and post-contrast T1, extracellular volume (ECV), and T2 maps of the myocardium in both healthy individuals and patients with HCM. Our findings demonstrate that by employing a newly-built 72-channel coil, parallel imaging using SENSE factors of up to 6 becomes feasible, thereby reducing the number of required BHs to 1 for native and post-contrast T1 maps, and 2 BHs for T2 maps (4 BHs in total). To assess the reliability and accuracy of the accelerated scans, repeatability and precision of T1 and T2 were evaluated in phantoms and healthy study participants, and compared against measurements obtained using the vendor-supplied 16-channel receive array coil. Additionally, we successfully demonstrated the applicability of the 72-channel coil accelerated protocol in quantifying native and post-contrast T1 and ECV in patients with HCM.

## Materials and methods

### Study participants

The *in vivo* studies were approved by the local ethical board committee and conducted according to the Declaration of Helsinki. All study participants provided written consent before inclusion (METC approval number NL71689.018.20). 20 healthy study participants (10 female) were prospectively included for the accelerated native T1 and T2 protocol. Recruitment of participants took place via public advertisements of the study. All 20 participants were healthy without a history of cardiovascular disease, had no contraindications to MRI examination (e.g., pacemaker, metal fragments, implants, arrhythmias, or claustrophobia) and all were non-smokers. In addition, we scanned 3 male HCM patients.

### MR methods

**Figure 1:**
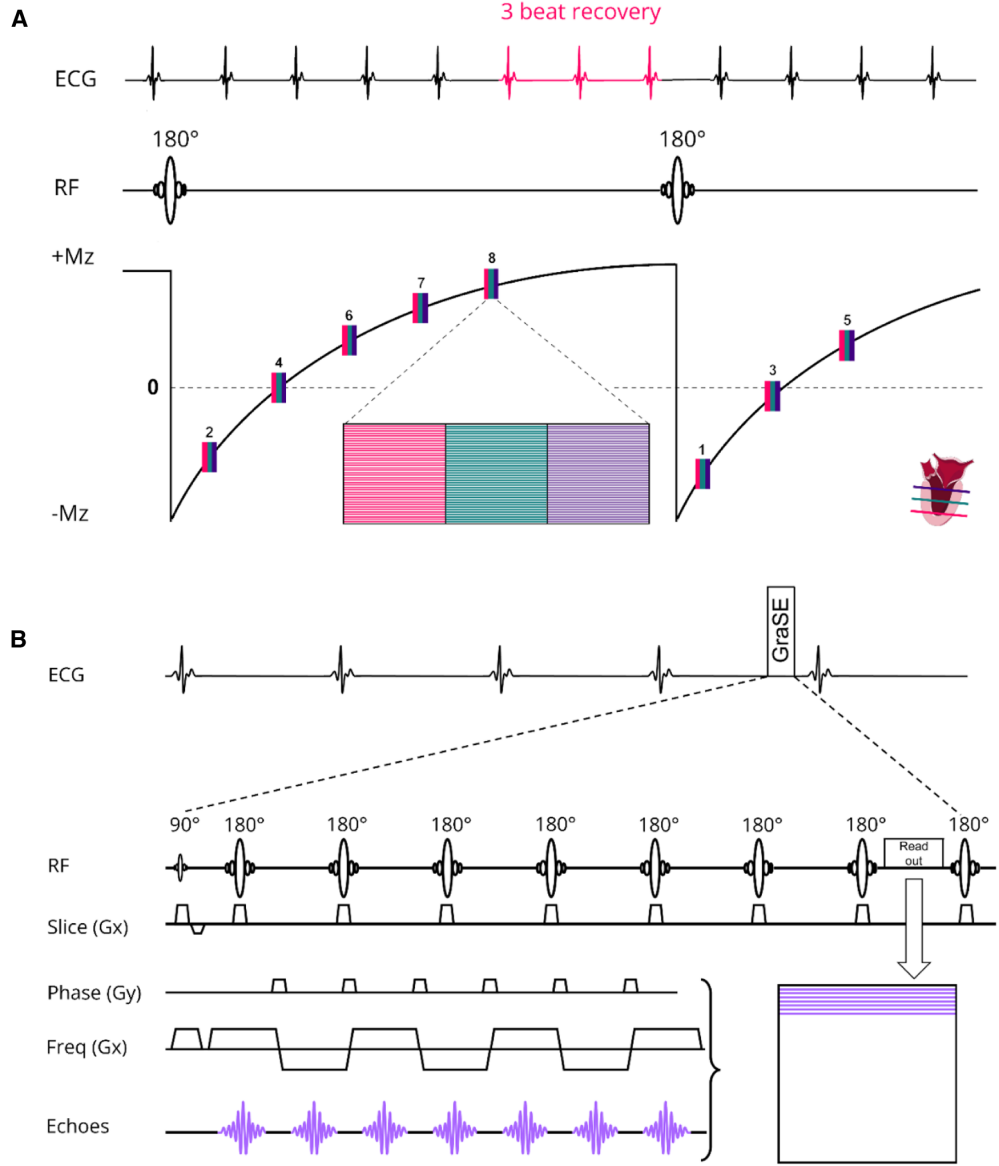
(**A**) native T1 modified Look locker (MOLLI) multislice sequence using shared 180° inversion pulses where the apical (red), mid-ventricular (green) and basal (deep purple) slices are acquired interleaved in a 5s(3s)3s scheme. For post-contrast T1 mapping we used a 4s(1s)3s(1s)2s scheme. (**B**) T2 Gradient-Spin-Echo (GraSE) sequence which combines a Turbo-Spin-Echo (TSE) with an echo planar imaging (EPI) readout. ECG, electrocardiogram; Mz, net magnetization; RF, radiofrequency pulse; Freq, frequency.

The scanning procedures were conducted using a 3 T MR scanner (Ingenia, Philips Healthcare, Best, The Netherlands) and involved the utilization of two distinct receive array coils: the vendor-supplied 16-channel receive array coil and the newly developed, smaller and lighter 72-channel receive array coil. It is important to mention that both coil arrays were used in conjunction with a 12-channel posterior array coil integrated into the patient table, resulting in a total of 28 and 84 receive channels, respectively. However, to prevent any potential confusion, we will refer to the coils as the 16-channel and 72-channel receive array coil throughout the manuscript. Prior research had shown that the noise correlation, covariance matrix, the coils sensitivity and signal-to-noise ratio (SNR) distribution of the 72-channel receive array coil were sufficient to maximize SNR and minimize the coil geometry factor (g-factor) (20). The design of the 72-channel receive array coil was described in detail previously (19). The MOLLI sequence for native T1 mapping employed the 5s(3s)3s scheme with two 180° inversion pulses and single-shot bSSFP read-outs per slice and BH. For post-contrast T1 mapping we used the 4s(1s)3s(1s)2s scheme. To enable an interleaved acquisition of multiple slices, we modified the MOLLI sequence to have a single inversion pulse for a multi-slice readout. We refer to this protocol as the shared inversion pulse interleaved MOLLI sequence (sipiMOLLI) and it is schematically depicted in Figure 1A. Because the different slices are measured sequentially, the inversion time for the different slices is slightly different, which was appropriately taken into account in the T1 fit. For T2 mapping a GraSE sequence was used which combines a Turbo-Spin-Echo (TSE) with an echo planar imaging (EPI) readout, as schematically depicted in Figure 1B. The train of spin echoes determined by the TSE factor equals the number of images used to generate the T2 maps. The EPI factor determines the number of k-lines that are sampled after each 180-degree pulse in the TSE train. With an increasing acceleration factor, the number of shots needed to complete k-space decreases, enabling the acquisition of multiple slices, fewer BHs, or shorter BH duration. Detailed sequence parameters of the T1 and T2 mapping sequence are listed in Table 1.

**Table 1:** Acquisition parameters sequences.

Parameters	T1 MOLLI	T2 GRaSE
Slice thickness/gap	8/16 mm	8/16 mm
TR/TE/Flip angel	3.6 ms/1.6 ms/20°	1 RR-interval/9.3 ms/90°
Field of View	300 × 300 mm	350 × 350 mm
Acquisition plane resolution	2.0 mm	2.0 mm
Reconstructed plane resolution	1.17 mm	1.22 mm
Trigger delay	Longest	Longest
MOLLI schemes	Native: 5s(3s)3s	n.a.
	Post: 4s(1s)3(s1s)2s	
TFE-factors	93 (S = 2)	n.a.
	46 (S = 4)	
	32 (S = 6)	
Inversion time (TI)^a^	141 ± 3 ms (S = 2)	n.a.
	219 ± 105 ms (S = 4)	
	169 ± 79 ms (S = 6)	
TSE-factor	n.a.	8
EPI-factor	n.a.	7
Echo spacing	n.a.	8.5
Total BH time^b^ (s)	33 s (S = 2)	45 s (S = 2)
	11 s (S = 4/6)	27 s (S = 4)
		21 s (S = 6)
BHs^c^, n	3 (S = 2)	3 (S = 2)
	1 (S = 4/6)	2 (S = 4/6)

^a^ Mean ± standard deviation.

^b^ Time based on heart frequency of 60 beats/min.

^c^ Based on clinically appropriate maximum breath-hold time of 15 s.

### MR experiments

#### Experiment 1: phantoms

**Figure 2:**
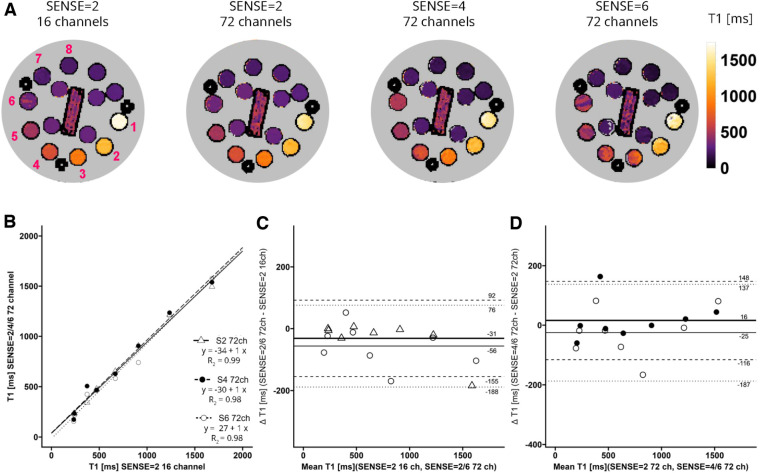
(**A**) representative T1 maps of the system phantom containing spheres with calibrated MnCl_2_ doped water acquired with SENSE = 2 and the vendor supplied 16-channel coil as well as SENSE = 2, 4, and 6 with the 72-channel coil. Lighter colors indicate higher values and darker colors indicate lower T2 values according to the color bar. The spheres numbered 1-8 have T1 values higher than 100 ms. (**B**–**D**) Linear regression plots and Bland-Altman plots comparing coils and SENSE acceleration factors. ms, milliseconds; S, SENSE; ch, channels.

A phantom for T1, T2 and Proton Density qMRI standardization (CaliberMRI) (21, 22) was used to compare T1 and T2 values derived from acquisitions with the vendor-supplied 16-channel receiver array coil and the 72-channel receiver array coil (Figure 2). The phantom contains multiple layers with spheres filled with calibrated NiCl_2_-doped water for T1 analysis and MnCl_2_ doped water for T2 analysis. First, acquisitions were made with the clinically used SENSE = 2 MOLLI-sequence and the 16- and 72-channel receive array coils. Secondly, scans were repeated with the sipiMOLLI sequence and SENSE = 2, 4, and 6 in conjunction with the 72-channel receive array coil. The phantom was positioned in a cabinet inside the MR scanner room a day before examination for temperature stabilization. Room temperature remained constant during measurements (20.1°C).

#### Experiment 2: healthy study participants

MRI scans of the healthy study participants were carried out with the 72-channel receive array coil. To begin with, standard survey scans were acquired to plan 3 short-axis (SAx) slices (apical, mid-ventricular, and basal) for subsequent T1 and T2 mapping. Next, a standard MOLLI scan on 3 slices with SENSE = 2 was acquired requiring 3 BHs, followed by sipiMOLLI on the same 3 slices with SENSE = 4 and 6, requiring 1 BH. After this, T2 measurements using the GraSE sequence were acquired using SENSE = 2, 4, and 6 requiring 3, 2, and 2 BHs, respectively.

#### Experiment 3: HCM patients

The HCM patients first received native T1 mapping with MOLLI with SENSE = 2 (3 BHs), and sipiMOLLI with SENSE = 4 (1 BH) in 3 short-axis (apical, mid-ventricular, and basal) slices. Subsequently, 0.2 ml/kg body weight of a gadolinium-based contrast agent (0.5 mmol/ml, DOTAREM®, Guerbet, France) was administrated via a cannula in the median cubital vein at an injection rate of 1 ml/s followed by a 15 ml saline flush. After approximately 15 min, post-contrast T1 mapping was performed using MOLLI with SENSE = 2 (3 BHs) followed by sipiMOLLI with SENSE = 4 (1 BH).

### Analysis

#### Qualitative analysis

Visual expert scoring of all slices, graded on a scale of 1–5 (1 = nondiagnostic, 2 = suboptimal, 3 = adequate, 4 = good, and 5 = excellent) were independently performed by 2 senior researchers (MR physicist and cardiologist with 15 + years expierence in cardiac MRI) with focus on the presence of artifacts.

#### Relaxometetry and ECV

T1 and T2 maps calculated in the phantom, healthy study participants and HCM patients were analyzed using the QMRITools toolbox (23) for Mathematica (version 13.1, Wolfram, Hanborough, UK). Subsequently, T1 and T2 relaxation time constants were calculated per voxel by fitting the appropriate exponential relaxation curves through the signal intensities as a function of inversion or echo time. To test for accuracy and precision in the phantom experiments, T1 spheres with a relaxation time of higher than 100 ms were included (i.e., spheres 1–8). For T2, spheres with a relaxation time between 20ms−200 ms (i.e., spheres 4–10) are in the range with expected myocardial T2 values (24). Native and post-contrast T1 and T2 maps acquired in healthy study participants and HCM patients were analyzed according to a recent consensus paper (25). In short, manually drawn region of interests (ROIs) covering the full LV-mass excluding the blood pool and papillary muscles were used to quantify native T1 and T2 values for the 3 slices combined per slice and according to the American Heart Association 16-segment model (5). For post-contrast T1, septal and blood pool ROIs were manually drawn avoiding papillary muscles in both the mid-ventricular and basal slice for validation. The ECV was calculated per voxel according to:

$$\begin{array}{rl} \mathrm{ECV} & =(1-\mathrm{Hct})\times [\left(R_{1,\;\mathrm{myocardium}\;\mathrm{post}}-R_{1,\;\mathrm{myocardium}\;\mathrm{native}}\right)\\ & \;/(R_{1,\;\mathrm{blood}\;\mathrm{post}}-R_{1,\;\mathrm{blood}\;\mathrm{native}})]\times 100\text{\%} \end{array}$$

where Hct is the blood hematocrit (L/l) value and R_1_ is the relaxation rate 1/T_1_. Since there were no blood samples accessible, the Hct value was approximated to be 0.4.

#### Statistical analyis

Statistical analysis was performed using the software packages R (version 4.2.2, The R Foundation for Statistical Computing, Vienna, AT) and Rstudio (version 2022.07.2 + 576, Posit, Boston, MA, USA). Statistical significance was accepted for *p*-values < 0.05. *P*-values were adjusted for multiple comparisons using the false discovery rate (FDR) method as proposed by Benjamini and Hochberg (26). Data were first tested for normality. Subsequently means and standard deviations (SD) or medians and interquartile ranges (IQR) are presented. For the phantom, pairwise comparisons were made using paired t-tests, linear regressions and Bland-Altman analyses. In the healthy study participants, analysis was extended with two 2-factor ANOVAs with PI-factor, slice and segment as factors to analyze slices and the 16-segmental model. Reviewer agreement was tested using a Wilcoxon signed rank, Chornbach’s Alpha (irr package, tolerance = 1) and Cohen’ Kappa test. Wilcoxon signed rank tests were used to compare the image quality scores of presence of artifacts between methods. Given the limited number of study participants, descriptive statistics were performed on the data of HCM patients.

## Results

### Study participants

Of the 20 healthy study participants 2–4 were not included in the analysis due to operator and/or post-processing errors during and/or after the MRI examination. Thus, 16 for T1 and 18 for T2 healthy study participants (8 female, 28 ± 5 years; body mass index, 22.4 ± 2.1 kg/m^2^) were included in the analysis. Measurements and analyses of the 3 HCM patients (3 males, 55 ± 14 years; body mass index, 27.6 ± 3.7 kg/m^2^) were successful. Demographic characteristics of healthy study participants and HCM patients are presented in Table 2.

**Table 2:** Characteristics of the study population.

	Healthy	HCM
Number of subjects	16–18	3
Percentage women (%)	50	0
Age (years)	28 ± 5^a^	55 ± 14^a^
Height (cm)	182 ± 28^a^	183 ± 6^a^
Weight (kg)	75 ± 13^a^	93 ± 19^a^
BMI	22.4 ± 2.1^a^	27.6 ± 3.7^a^
Body Surface Area (m^2^)	1.9 ± 0.2^a^	2.2 ± 0.26^a^
Heart rate (beats/min)	59 ± 10^a^	65 ± 10^a^

HCM, hypertrophic cardiomyopathy.

^a^ Mean ± standard deviation.

### Phantom

T1 values of the phantom are shown in Figure 2A. Linear regression and Bland-Altman analyses (Figures 2B–D) revealed good agreement between maps acquired with the 72-channel receive array coil and SENSE = 2, 4, and 6 compared to the maps acquired with the 16-channel receiver array coil and SENSE = 2 (Figures 2B–D). Linear regression resulted in *R*^2^ = 0.99-0.98 for SENSE = 2, 4, and 6. The Bland-Altman analysis in Figure 2C revealed no significant bias between T1 acquired with the 16-channel receive array coil and the accelerated scans with bias values: SENSE = 2: T1_bias_ = −31 ± 124 ms, *p* = 0.300, SENSE = 4: T1_bias_ = −15 ± 150 ms, *p* = 0.590, SENSE = 6: T1_bias_ = −56 ± 132 ms, *p* = 0.050). Similarly, when SENSE = 2 on the 72-channel receive array coil was used as a comparator (Figure 2D), also no significant bias was observed for SENSE = 4: T1_bias_ = 16 ± 132 ms, *p* = 0.523 and SENSE = 6: T1_bias_ = −25 ± 162 ms, *p* = 0.424.

**Figure 3:**
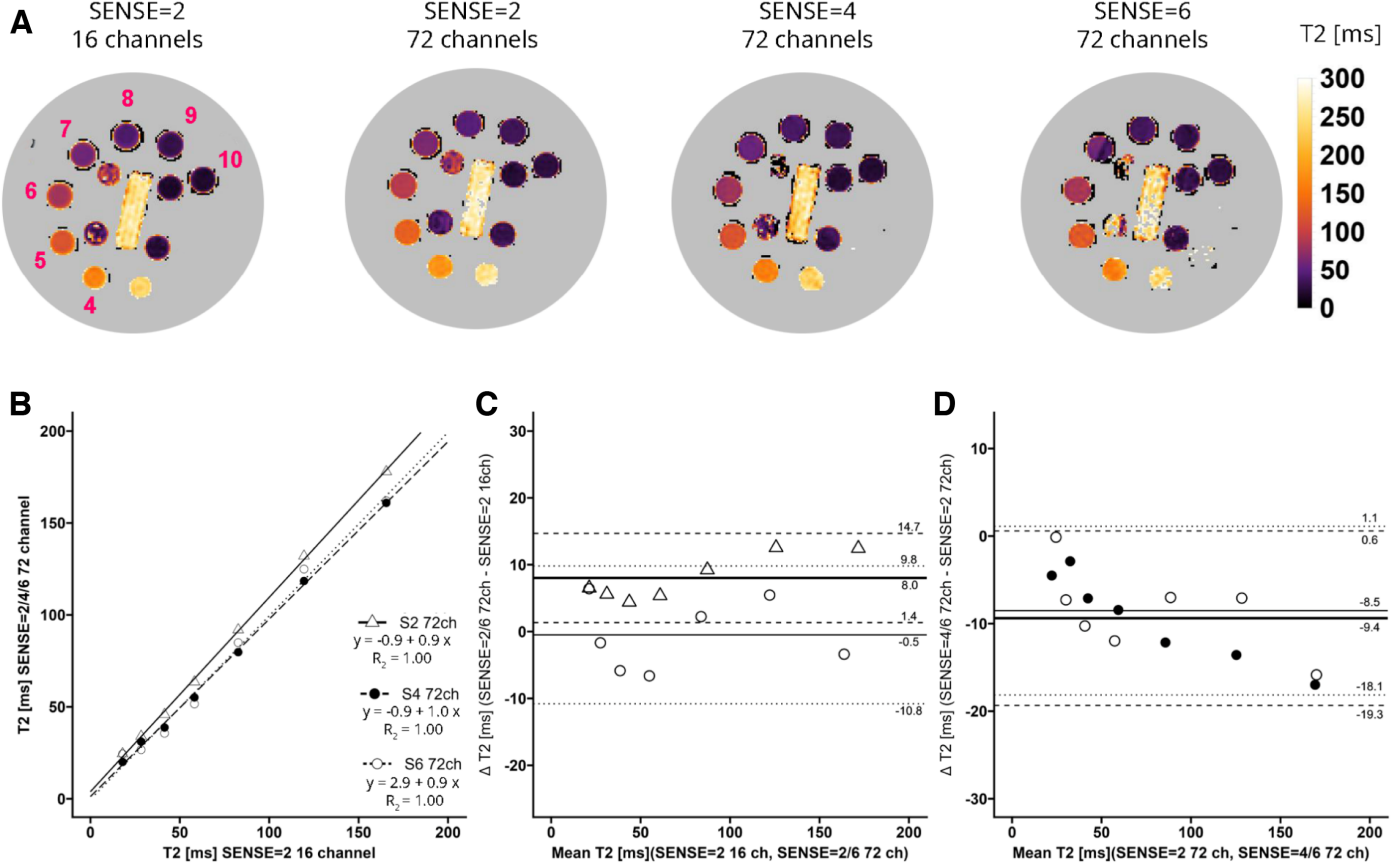
(**A**) representative T2 maps of the system phantom containing spheres with calibrated MnCl_2_ doped water acquired with SENSE = 2 and the vendor supplied 16-channel coil as well as SENSE = 2, 4, and 6 with the 72-channel coil. Lighter colors indicate higher values and darker colors indicate lower T2 values according to the color bar. The spheres numbered 4–10 have T2 values in a the relevant range of 20 ms–200 ms. (**B**–**D**) Linear regression plots and Bland-Altman plots comparing coils and SENSE acceleration factors. ms, milliseconds; S, SENSE; ch, channels.

For T2 (Figure 3A) linear regression between maps acquired with the 16-channel receiver array coil versus the 72-channel receiver array coil resulted in *R*^2^ = 1.00-0.99, for SENSE = 2, 4, and 6. Figure 3B shows that there was a systemic bias between acquisitions with the 16-channel receive array coil and SENSE = 2 as compared to those with the 72-channel receive array coil and SENSE = 2 (T2_bias_ = 8.0 ± 6.7 ms, *p* < 0.001), but not for SENSE = 4 (T2_bias_ = −1.4 ± 5.4 ms, *p* = 0.239) and SENSE = 6 (T2_bias_ = −0.5 ms ± 10.3 ms, *p* = 0.813). When the 72-channel receive array coil with SENSE = 2 was used as comparator (Figure 3D), there was a significant bias of SENSE = 4 (T2_bias_ = −9.4 ± 10.0 ms, *p* = 0.003) and SENSE = 6 (T2_bias_ = −8.5 ± 9.6 ms, *p* = 0.004—Figure 3D).

### Healthy study participants

**Figure 4:**
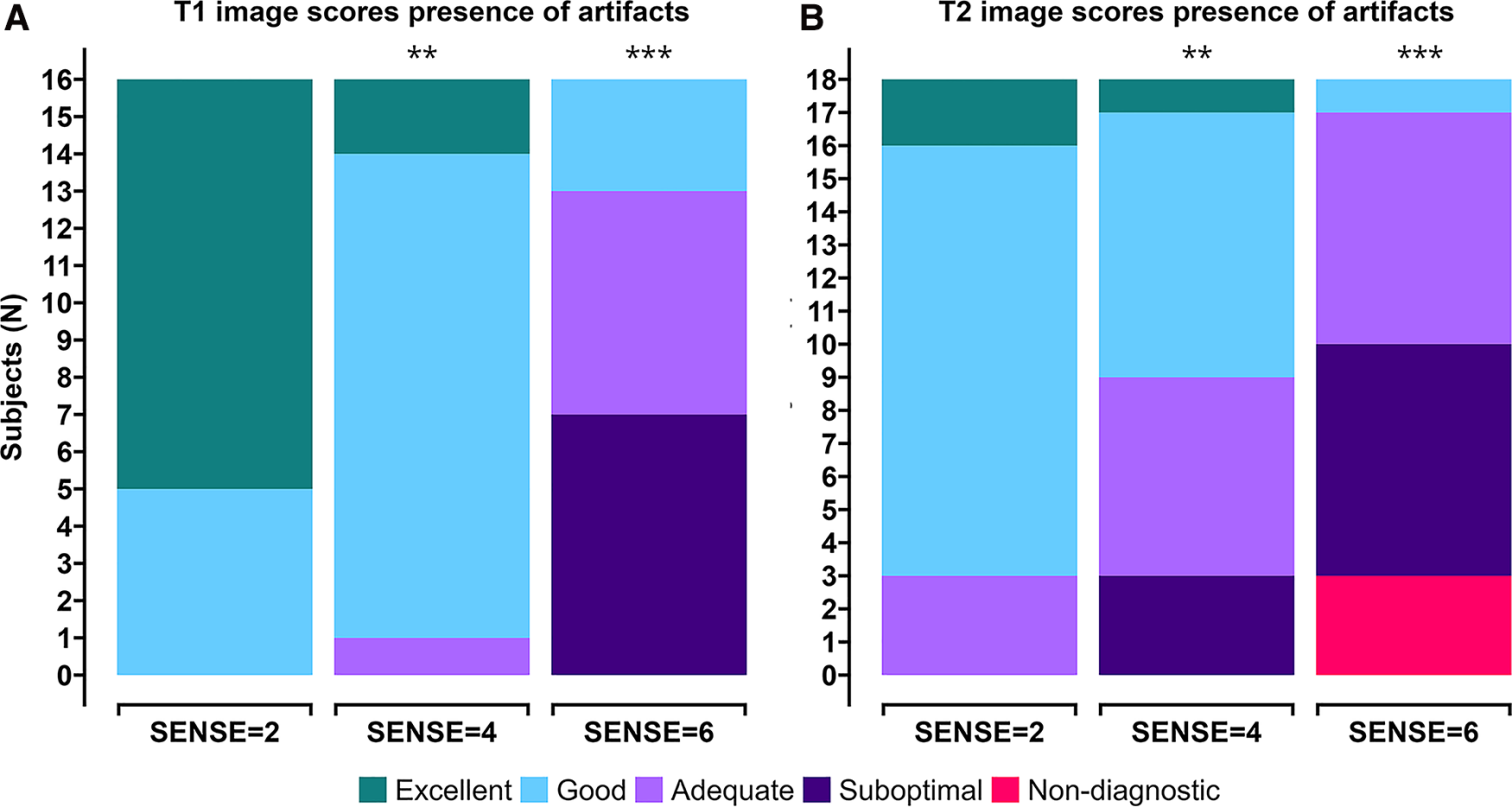
Stacked (**A**) T1 qualitative scoring and (**B**) T2 qualitative scoring bar charts of combined averaged image quality at apical, mid-ventriclar and basal level per subject. Colors depict an image quality score of excellent (green), good (light blue), adequate (light purple), suboptimal (dark purple), or non-diagnostic (red) in a grading criteria based on presence of artifacts. *p < 0.05, **p < 0.01, ***p < 0.001.

Image quality scores between 2 experts were similar for all criteria for all scans for both T1 (*p* = 0.71, 96.3% agreement, Kappa: 0.4) and T2 (*p* = 0.099, 94.4% agreement, Kappa: 0.5). Combined averaged scores by both experts in each criterion were used for further analysis. Figures 4A,B shows the T1 and T2 qualitative scoring of the apical, mid-ventriclar and basal slices combined per subject. For the T1 reference scan, all data received good to excellent scores. These scores were affected slightly using SENSE = 4 for which only 1 participant received an image artifact score of 3 (adequate) with no suboptimal or non-diagnostic score. With SENSE = 6, 9/16 participants scored 3–4 (adequate-good) and 7/16 participants received an image artifact score of 2 (suboptimal). Median (Md.) score for SENSE = 2 was 5. Median scores were lower for SENSE = 4 (Md. = 4.25, effect size *r* = 0.385, *p* = 0.002) and SENSE = 6 (Md. = 2.75, *r* = 0.736, *p* < 0.001).

For the T2 reference scan, all data received adequate to excellent scores. The results using SENSE = 4 were somewhat different: a small decrease in image quality scores was observed. Nevertheless, 15/18 participants received adequate-excellent scores, whereas 3/18 participants received suboptimal scores for image artifacts. The use of 6-fold acceleration further affected the artifact scores, where 8/18 scored adequate-good and 10/18 subjects scored non-diagnostic/suboptimal. Median score for SENSE = 2 was 4.08. Median scores were lower for SENSE = 4 (Md. = 3.42, effect size *r* = 0.245, *p* = 0.007) and SENSE = 6 (Md. = 2.50, *r* = 0.551, *p* < 0.001).

**Figure 5:**
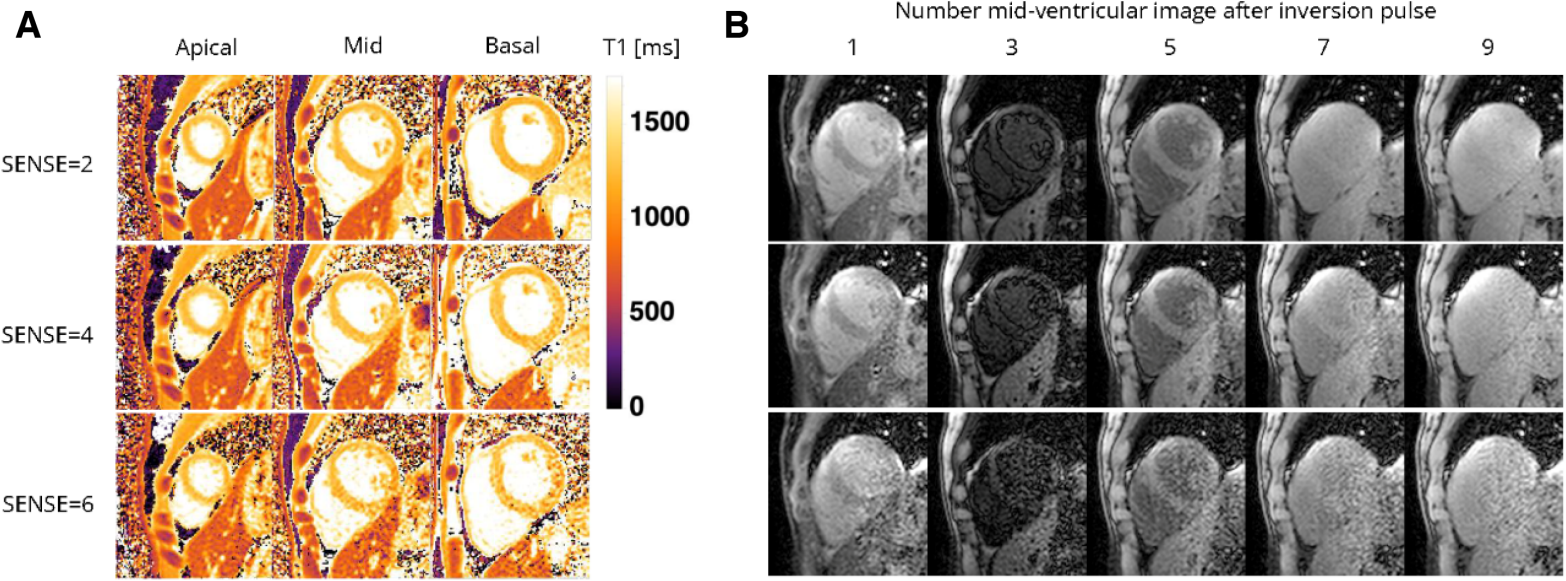
Representative (**A**) T1 maps and (**B**) T1-weighted images (time points 1, 3, 5, and 7 in an inversion train of 10) of a healthy female volunteer acquired with the 72-channel array coil using the single BH shared-inversion pulse interleaved MOLLI with SENSE = 2 and 6. Lighter colors indicate higher T1 values and darker colors indicate lower T1 values according to the color bar. ms, milliseconds.

**Figure 6:**
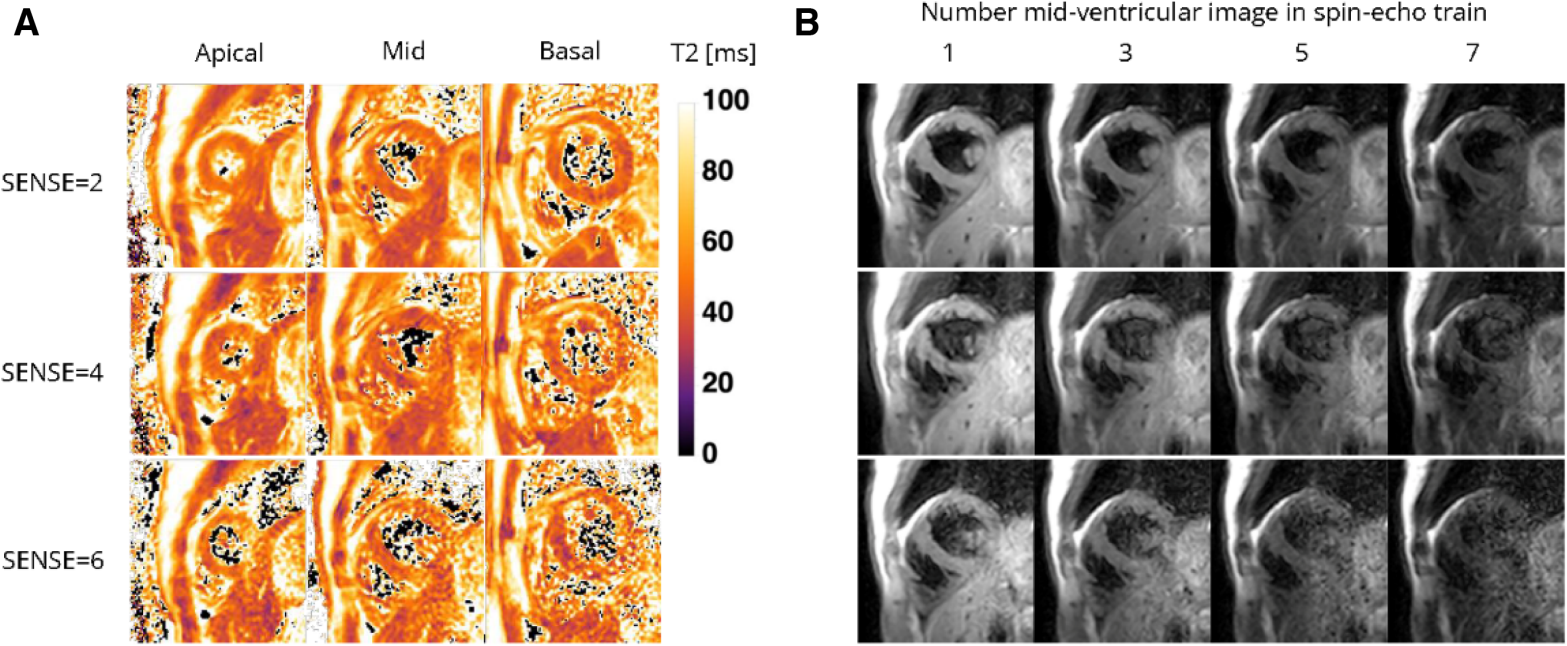
Representative (**A**) T2 maps and (**B**) T2-weighted images (echo 1, 3, 5, and 7 in spin-echo train of 8) of a healthy female volunteer acquired with the 72-channel coil using a gradient-spin-Echo (graSE) sequence with SENSE = 2, 4, and 6. Lighter colors indicate higher T2 values and darker colors indicate lower T2 values according to the color bar. ms, milliseconds.

**Figure 7:**
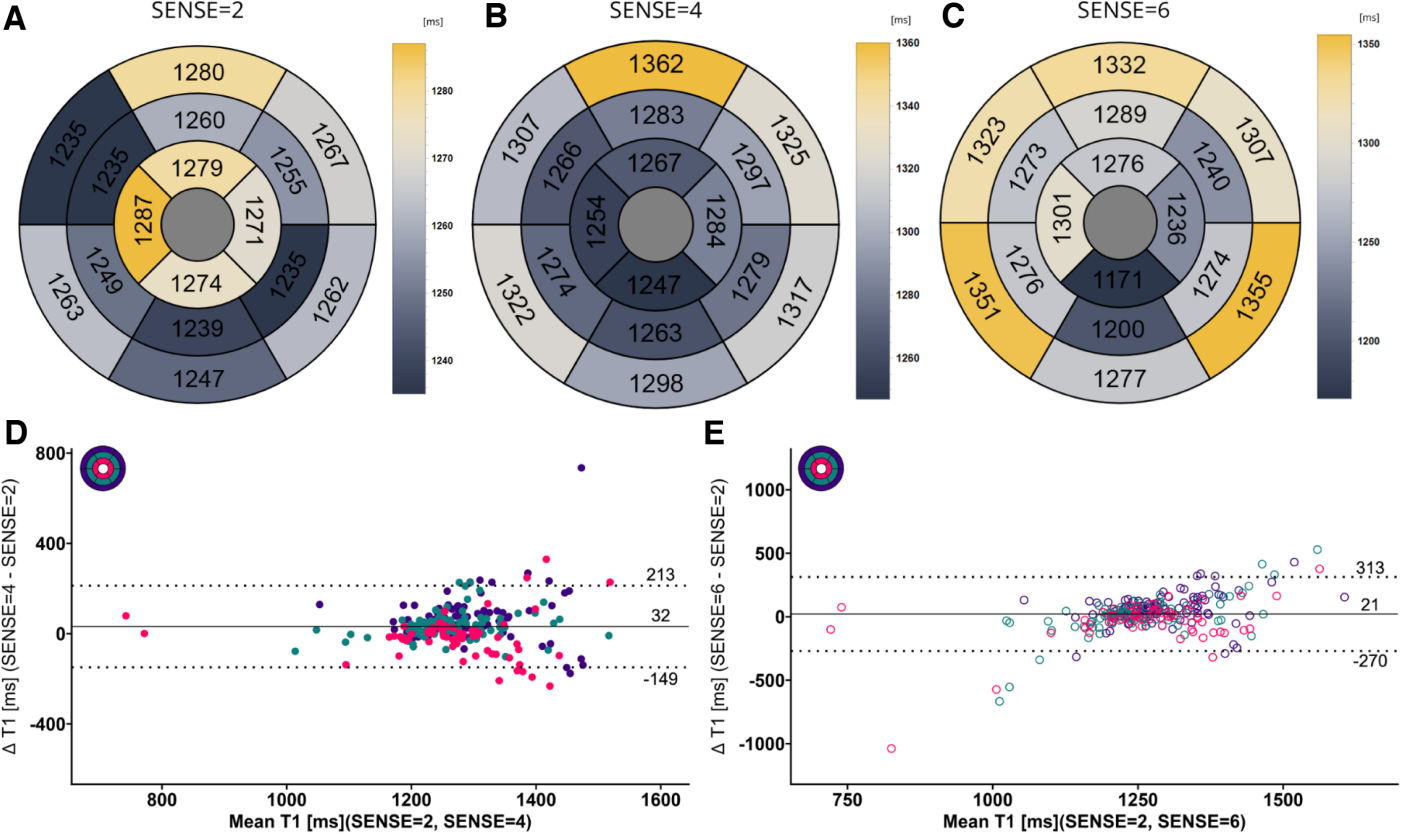
(**A**–**C**) mean native T1 values per segment according to the 16-segment AHA model for SENSE = 2, 4, and 6. (**D**,**E**) Bland-Altman plots comparing segment-wise native T1 differences for SENSE = 4 and SENSE = 6. Confidence intervals of the mean difference in the Bland-Altman plots are presented in Table 4. Color code of the slices in the Bland-Altman plots: red = apical, green = mid-ventricular, deep purple = basal. ms, milliseconds.

**Figure 8:**
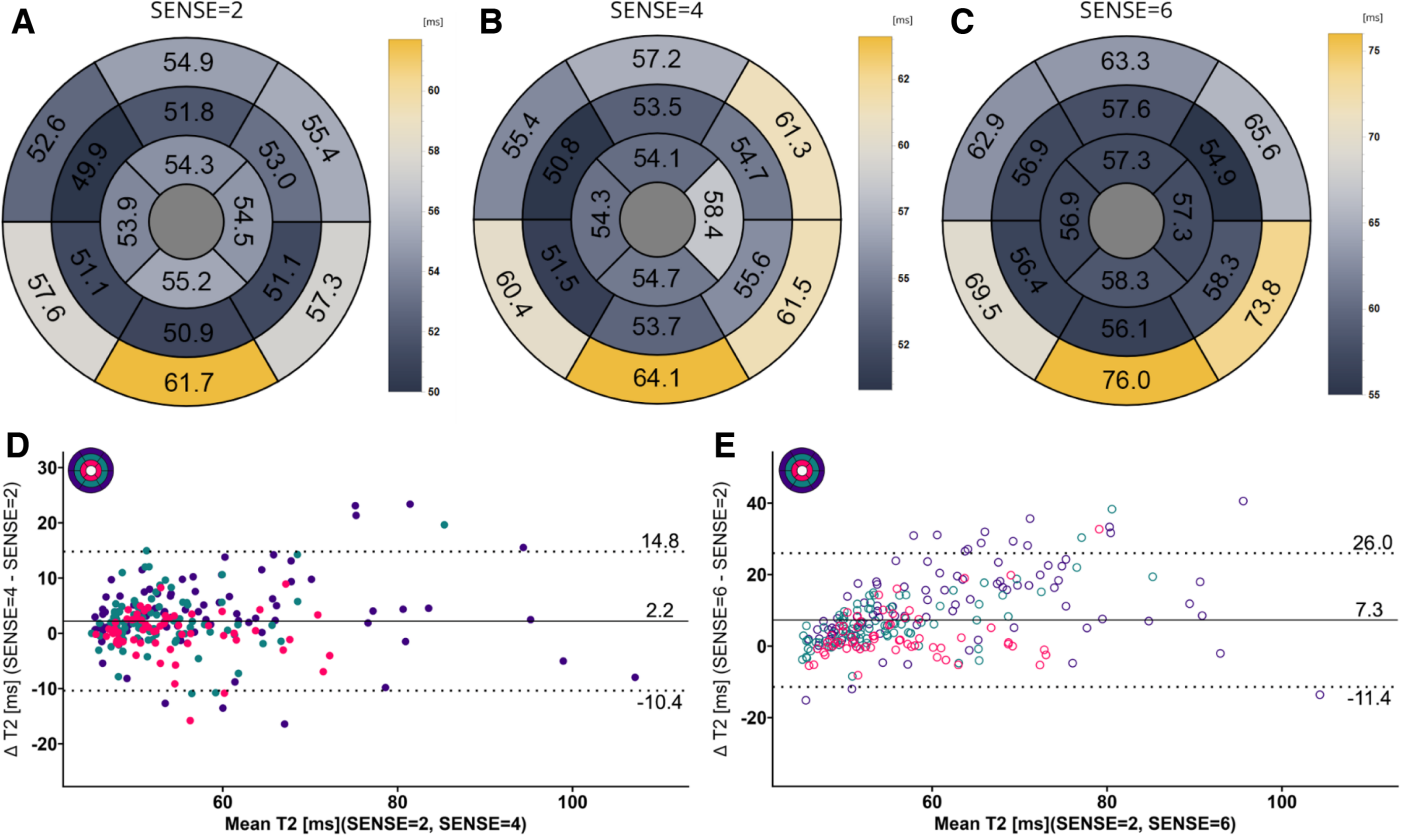
(**A**–**C**) mean T2 values per segment according to the 16-segment AHA model for SENSE = 2, 4, and 6. (**D**,**E**) Bland-Altman plots comparing segment-wise T2 differences for SENSE = 4 and SENSE = 6. Confidence intervals of the mean difference in the Bland-Altman plots are presented in Table 4. Color code of the slices in the Bland-Altman plots: red = apical, green = mid-ventricular, deep purple = basal. ms,  milliseconds.

Figures 5, 6 contain representative T1 and T2 maps acquired with the various protocols. Global, slice and segmental native T1 and T2 values are presented in Table 3 and Figures 7A–C, 8A–C. Agreements among the different SENSE factors are presented in Table 4 and in the Bland–Altman plots in Figures 7D,E, 8D–E for global, slice-based and segment-based analysis.

**Table 3:** Descriptive data and pairwise comparisons for global, slice (basal, mid-cavity, apical) and for segments (16 AHA model) of myocardial native T1 and T2 values.

PI-factor	SENSE = 2				SENSE = 4						SENSE = 6					
	T1 mean	SD	T2 mean	SD	T1 mean	SD	*p*-value	T2 mean	SD	*p*-value	T1 mean	SD	*p*-value	T2 mean	SD	*p*- value
Global	1245	51	51.5	5.0	1270	47	<0.001***	53.6	5.7	<0.001***	1276	57	<0.001***	57.7	6.7	<0.001***
Basal	1241	45	53.0	6.8	1296	38	<0.001***	57.00	8.1	<0.001***	1317	62	<0.001***	62.9	8.6	<0.001***
1	1280	110	54.9	17.4	1362	142	0.173	57.2	17.2	0.729	1332	73	0.148	63.3	14.4	**0.011** *
2	1235	81	52.6	9.4	1307	94	<0.001***	55.4	11.1	0.003***	1323	134	**0.010** **	62.9	12.5	<0.001***
3	1263	46	57.6	9.9	1322	74	**0.006** **	60.4	10.5	0.178	1351	106	**0.006** **	69.5	12.8	<0.001***
4	1247	73	61.7	15.5	1298	73	**0.047** *	64.1	16.7	0.795	1277	125	0.512	76.0	21.0	**0.005** **
5	1262	91	57.3	15.8	1317	87	**0.013** *	61.5	15.4	0.258	1355	138	**0.009** **	73.8	22.1	<0.001***
6	1267	84	55.4	8.5	1325	89	0.120	61.3	12.0	**0.006** **	1307	94	0.304	65.6	15.0	**0.009** **
Mid	1235	48	50.8	5.4	1261	55	**0.003** **	52.1	4.6	**0.044** *	1252	52	**0.035** *	55.8	7.1	***p < 0.001
7	1260	57	51.8	7.6	1283	71	0.297	53.5	12.2	0.702	1289	91	0.297	57.6	12.0	**0.004** **
8	1235	39	49.9	4.3	1266	42	**0.011** *	50.8	3.3	1.000	1273	169	0.498	56.9	10.7	**0.006** **
9	1249	49	51.1	5.3	1274	49	**0.020** *	51.5	4.4	1.000	1276	93	0.265	56.4	7.1	**0.008** **
10	1239	109	50.9	6.7	1263	123	0.262	53.7	7.1	0.138	1200	188	0.387	56.1	10.8	0.065
11	1235	81	51.1	5.7	1279	108	0.063	55.6	7.5	**0.009** **	1274	203	0.547	58.3	13.4	**0.025** *
12	1255	95	53.0	8.1	1297	106	0.128	54.7	7.9	0.585	1240	168	0.739	54.9	12.7	0.038*
Apical	1258	112	52.5	6.8	1250	77	0.720	53.5	6.4	0.103	1254	94	0.720	56.7	6.2	**0.001** **
13	1279	81	54.3	6.6	1267	84	0.904	54.1	5.8	1.000	1276	64	0.904	57.3	6.7	0.053
14	1287	74	53.9	5.5	1254	66	0.164	54.3	5.9	1.000	1301	146	0.633	56.9	3.9	0.138
15	1274	171	55.2	8.5	1247	153	0.264	54.7	7.3	1.000	1171	293	0.264	58.3	13.1	0.717
16	1271	178	54.5	9.4	1284	182	0.685	58.4	19.9	0.843	1236	214	0.616	57.4	10.8	0.272

Data are presented as the mean ± standard deviation. PI, parallel imaging; ref, reference.

* *p* < 0.05.

** *p < *0.01.

*** *p* < 0.001.

**Table 4:** Bland-Altman analyses for global, slice (basal, mid-cavity, apical combined) and segments (16 AHA model combined) of myocardial native T1 and T2 values.

Ref. group	SENSE = 2					
PI-factor	SENSE = 4			SENSE = 6		
** **	Mean bias ± LoA	Conf.	*p*-value	Mean bias ± LoA	Conf.	*p*-value
T1						
Global	25 ± 41	11.22^a^	<0.001***	31 ± 48	12.99^a^	<0.001***
Slice	25 ± 87	12.86^a^	<0.001***	30 ± 103	15.25^a^	<0.001***
16 segments	32 ± 181	11.37^a^	<0.001***	21 ± 292	18.34^a^	**0**.022*
T2						
Global	2.1 ± 3.1	0.84^a^	<0.001***	6.2 ± 5.0	1.36^a^	<0.001***
Slice	2.1 ± 5.0	0.74^a^	<0.001***	6.4 ± 9.7	1.44^a^	<0.001***
16 segments	2.2 ± 12.6	0.80^a^	<0.001***	7.4 ± 18.7	0.46^a^	<0.001***

Data are presented as the mean bias, 95% limits of agreements of the mean bias and standard error*t as confidence of the mean difference. LoA, limits of agreement; PI, parallel imaging; ref, reference.

^a^ systematic bias—line of equality (0) outside the confidence intervals of the mean difference.

* *p* < 0.05.

** *p < *0.01.

*** *p* < 0.001.

Global mean native T1 values were different between SENSE = 2 (T1 = 1245 ms ± 51 ms), SENSE = 4 (T1 = 1270 ms ± 47 ms, *p* < 0.001) and SENSE = 6 (T1 = 1276 ms ± 57 ms, *p* < 0.001) resulting in T1 overestimation of 25 ± 41 ms for SENSE = 4 and 31 ± 48 ms for SENSE = 6. The 2-factor ANOVA with PI-factor and slice as factors revealed an interaction effect (*p* = 0.047) with different native T1 values on basal and mid-ventricular but not on an apical level between SENSE = 2 (basal: T1 = 1241 ± 45 ms, mid: T1 = 1235 ms ± 48 ms, apical: T1 = 1258 ms ± 112 ms), SENSE = 4 (basal: T1 = 1296 ms ± 38 ms, *p* < 0.001, mid: T1 = 1261 ms ± 55 ms, *p* = 0.003, apical: T1 = 1250 ms ± 77 ms, *p* = 0.720) and SENSE = 6 (basal: T1 = 1317 ± 62 ms, *p* < 0.001, mid: T1 = 1252 ± 52 ms, *p* = 0.035, apical: T1 = 1254 ± 94 ms, *p* = 0.720). We found that slices-averaged native T1 values were overestimated by 25 ms ± 87 ms using SENSE = 4 and 30 ms ± 103 ms using SENSE = 6. On the segment level, differences were found for segments 2 (*p* < 0.001), 3 (*p* = 0.006), 4 (*p* = 0.047), 5 (*p* = 0.013), 8 (*p* = 0.011) and 9 (*p* = 0.020) for SENSE = 4 and with segments 2, (*p* = 0.010), 3 (*p* = 0.006) and 5 (*p* = 0.009) for SENSE = 6. Segments-averaged T1 values were overestimated by 32 ms ± 181 ms for SENSE = 4 and 21 ms ± 292 ms for SENSE = 6.

Mean global heart T2 values, were different for SENSE = 2 (T2 = 51.5 ± 5.0 ms), SENSE = 4 (T2 = 53.6 ± 5.7 ms, *p* < 0.001) and SENSE = 6 (T2 = 57.7 ± 6.7 ms, *p* < 0.001) resulting in a T2 overestimation of 2.1 ± 3.1 ms for SENSE = 4 and 6.2 ± 5.0 ms for SENSE = 6. The 2-factor ANOVA with PI-factor and slice as factors revealed an interaction effect (*p* = 0.024) with different T2 values on all levels except apical for SENSE = 4 between SENSE = 2 (basal: T2 = 53.0 ± 6.8 ms, mid: T2 = 50.8 ± 5.4 ms, apical: T2 = 52.5 ± 6.8 ms), SENSE = 4 (basal: T2 = 57.0 ± 8.1 ms, *p* < 0.001, mid: 52.1 ± 4.6 ms, *p* = 0.044, apical: T2 = 53.5 ± 6.4 ms, *p* = 0.103) and SENSE = 6 (basal: T2 = 62.9 ± 8.6 ms, *p* < 0.001, mid: 55.8 ± 7.1 ms, *p* < 0.001, apical: T2 = 56.7 ± 6.2 ms, *p* = 0.001). We found that slices-averaged native T2 values were overestimated by 2.1 ± 5.0 ms using SENSE = 4 and 6.4 ± 9.7 ms using SENSE = 6. On the segment level, differences were found for segments 2 (*p* = 0.003),6 (*p* = 0.006) and 11 (*p* = 0.009) for SENSE = 4 and with segments 1 (*p* = 0.011), 2 and 3 (*p* < 0.001), 4 (*p* = 0.005), 5 (*p* < 0.001), 6 (*p* = 0.009), 7 (*p* = 0.004), 8 (*p* = 0.006), 9 (*p* = 0.008), 11 (*p* = 0.025) and 12 (*p* = 0.038) for SENSE = 6. Segments-averaged T1 values were overestimated by 2.2 ± 12.6 ms for SENSE = 4 and 7.4 ± 18.7 ms for SENSE = 6.

### Hypertrophic cardiomyopathy patients

**Figure 9:**
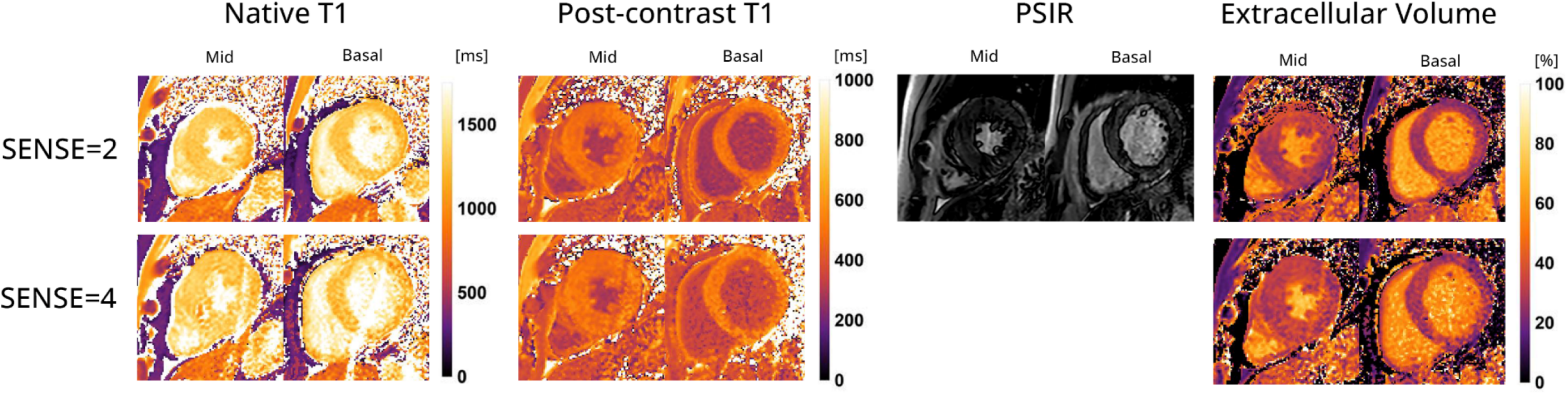
Representative native, post-contrast T1 maps, late gadolinium enhancement (LGE) with phase sensitive inversion recovery reconstruction (PSIR) images and extracellular volume (ECV) maps of a hypertrophic cardiomyopathy patient with the 72-channel array coil using a 3 BH MOLLI with SENSE = 2 compared to a single BH shared-inversion pulse interleaved MOLLI with SENSE = 4. Lighter colors indicate higher values and darker colors indicate lower values according to the color scales on the right.

Figure 9 displays the native and post-contrast T1 and ECV maps of a representative HCM patient using either the SENSE = 2 (3BH) or SENSE = 4 (1BH) protocols. Corresponding values at different cardiac slice locations are given in Table 5. In the mid-ventricular slice, native T1 values using SENSE = 2 vs. SENSE = 4 were T1 = 1366 ± 138 ms vs. T1 = 1417 ± 265 ms for subject 1, T1 = 1234 ± 158 ms vs. T1 = 1100 ± 405 ms for subject 2 and T1 = 1483 ± 404 ms vs. T1 = 1379 ± 173 ms for subject 3. Post-contrast T1 values were T1 = 437 ± 42 ms vs. 469 ± 54 ms for subject 1, T1 = 458 ± 49 ms vs. T1 = 597 ± 139 ms for subject 2 and T1 = 466 ± 48 ms vs. T1 = 498 ± 47 ms for subject 3. ECV values were 49.2 ± 7.8% vs. 48.2 ± 9.3% for subject 1, 31.5 ± 5.6% vs. 25.4 ± 11.3% for subject 2, and 32.0 ± 5.6% vs. 32.6 ± 4.9% for subject 3.

**Table 5:** Myocardial native, post-contrast T1 and extracellular volume values in hypertrophic cardiomyopathy patients.

Slice	Mid-ventricular		Basal	
Method	SENSE = 2	SENSE = 4	SENSE = 2	SENSE = 4
Subject 1				
Native T1 (ms)	1366 ± 138	1417 ± 265	1394 ± 194	1470 ± 274
Post-contrast T1 (ms)	437 ± 42	469 ± 54	422 ± 47	465 ± 98
ECV (%)	49.2 ± 7.8	48.2 ± 9.3	52.0 ± 10.0	50.6 ± 12
Subject 2				
Native T1 (ms)	1234 ± 158	1100 ± 405	1062 ± 206	1150 ± 419
Post-contrast T1 (ms)	458 ± 49	597 ± 139	419 ± 189	476 ± 173
ECV (%)	31.5 ± 5.6	25.4 ± 11.3	36.1 ± 10.0	37.0 ± 14.9
Subject 3				
Native T1 (ms)	1483 ± 404	1379 ± 173	1309 ± 123	1363 ± 337
Post-contrast T1 (ms)	466 ± 47.7	498 ± 47	473 ± 55	538 ± 182
ECV (%)	32.0 ± 6.5	32.6 ± 4.9	30.0 ± 7.0	30.3 ± 9.9

Data are presented as mean ± standard deviation.

In the basal slice, native T1 values using SENSE = 2 vs. SENSE = 4 were T1 = 1394 ± 194 ms vs. T1 = 1470 ± 274 ms for subject 1, T1 = 1062 ± 206 ms vs. T1 = 1150 ± 419 ms for subject 2 and T1 = 1309 ± 123 ms vs. T1 = 1363 ± 337 ms for subject 3 respectively. For post-contrast T1 values were T1 = 422 ± 47 ms vs. T1 = 465 ± 98 ms for subject 1, T1 = 419 ± 189 ms vs. T1 = 476 ± 173 ms for subject 2 and T1 = 473 ± 55 ms vs. T1 = 538 ± 182 ms for subject 3. ECV values were 52.0 ± 10.0% vs. 50.6 ± 12.0% for subject 1, 36.1 ± 10.0%, vs. 37.0 ± 14.9% for subject 2, and 30.0 ± 7.0% vs. 30.3 ± 9.9% for subject 3.

## Discussion

In this study, we showed that a multi-slice cardiac native and post-contrast T1, ECV, and T2 mapping protocol is feasible in as few as 4 BHs using a SENSE accelerated protocol facilitated by a newly developed 72-channel receive array coil. Phantom experiments revealed that the proposed approach achieved good accuracy and precision using SENSE = 4 and 6 compared with standard MOLLI and GraSE sequences. Qualitative scoring revealed a modest decrease using SENSE = 4 in image quality, which declined more with SENSE = 6. Global and slice myocardial native T1 and T2 values were slightly inferior compared to conventional 3 BH MOLLI and 3 BH GraSE in terms of accuracy and precision, though the majority of the segmental T1 and T2 values were statistically equivalent between SENSE = 2 and 4 (native T1: 10/16 segments = 63%, T2: 13/16 segments = 83%). Native and post-contrast T1 measurements and ECV quantification using SENSE = 4 in HCM patients were successful.

Image quality judged by the presence of artifacts revealed diminishing quality with higher acceleration factors. However, for native T1, even with SENSE = 6 none of the images were considered diasgnostically unusable. On the other hand, for T2, qualitative results were inferior to T1. This is likely due to the fact that the relatively long echo train in the GRaSe sequence makes the sequence more susceptible to movement artifacts. This is more problematic at higher acceleration factors when the number of measured k-space lines becomes very low and any motion-corrupted k-line therefore has a significant impact on image quality. The estimation of native T1 and T2 values was generally found to be slightly overestimated on a global, slice, and 16-segment level. While obtaining precise ground truth values *in vivo* can be challenging, we evaluated the systematic biases in T1 and T2 measurements with acceleration as a means to assess their accuracy. The bias values for T1 and T2 were slightly higher as compared to those reported in previous studies focusing on repeatability (27–29), but they still fall within a range that allows for the detection of subtle changes in these parameters associated with diseases (1).

Despite being limited to descriptive statistics in the HCM patients, it appears that the post-contrast T1 and ECV maps exhibited only slight variations which was also shown in a regular single-slice readout MOLLI SENSE = 4 study (30). Notably, subject 1 displayed elevated ECV values, which remained consistent between the un-accelerated and accelerated scans. These values, although still within the upper normal range based on clinical standards, indicate a potential deviation from normal levels (1, 31). Patient 2 demonstrated clinically plausible ECV values, albeit with higher standard deviations (SDs) in post-contrast T1 and ECV measurements. This higher SD can likely be attributed to the patient’s large body circumference, as indicated by a BMI of 31.9 kg/m^2^. A higher body circumference can impact the SNR due to the reduced sensitivity of the 72-channel receive array coil as the distance to the heart increases. The post-contrast T1 and ECV values and maps of patient 3 demonstrated close agreement with clinical values and exhibited small SDs in both values, as illustrated in Figure 9. This finding adds to our confidence in the effectiveness of our approach and its potential for practical application.

In addition to the acceleration factor, variances may also arise from subtle patient motion between BHs or differences in BH depth, leading to partial volume effects. Partial volume effects can significantly influence T1 and T2 values, particularly when adjacent structures like blood and pericardial fat are involved. Native T1 and T2 discrepancies were specifically observed in the basal slice and segments for both SENSE = 4 and SENSE = 6. This supports the suspicion that residual cardiac motion occurred during late diastole in the basal segments (32). Here we followed the current vendor supplied methods. Although currently there are no recommendations on acquisition resolution and interpolation (25) higher resolution imaging facilitated by accelerated imaging could help to decrease partial volume and inflow effects.

Some studies indicated the presence of T2 segmental variability in short-axis (SAx) and long-axis (LAx) in non-accelerated acquisitions specifically in the apical segments (24, 33). Several factors were considered as potential causes, including myocardial thickeness, partial volume effects, and artifacts caused by air from the lungs. However, when taking into account diastolic velocities, which are more noticeable in younger adults (34) as observed in the present study, it becomes plausible that residual diastolic movement in the basal region was present. This could be attributed to superior diastolic compliance (35) and lower vascular and ventricular stiffness (36). Automatic nonrigid motion correction and pixel-wise fitting (24) may resolve this problem (37).

The current accelerated approach guarantees that the duration of the T1 and T2 acquisitions is short enough to fit within the diastolic phase, even in the presence of higher heart rates (38). However, it is still recommended to consider increasing the acceleration factor for patients experiencing tachycardia and/or arrhythmias, as this can help reduce the acquisition time for each shot and decrease blurring or partial volume artifacts in the images.

In this study, we utilized the standard vendor-supplied SENSE method to accelerate the acquisitions, acknowledging that this approach is subject to g-factor considerations (39). The notable advantage of this approach is that it does not necessitate major sequence modifications or offline reconstruction, making it readily applicable in routine patient imaging. The present configuration of our coil design, consisting of 72 coil elements, restricts the achievable acceleration to approximately 6-fold. However, we believe that further optimization of the coil design is possible by incorporating additional coil elements in the posterior and lateral regions. This enhancement would improve coverage and help reduce g-factor noise amplification (20).

To achieve additional acceleration, one can consider embracing significant sequence modifications and implementing offline reconstruction techniques. This can be accomplished through the utilization of multi-band imaging techniques, 2D/3D sparse sampling schemes, and model-based reconstruction methods, in conjunction with a 72- or even higher-channel coil (8–11). In addition to enabling faster imaging, these approaches have the potential to reduce sensitivity to motion. For instance, studies have demonstrated that radial sampling schemes are inherently less affected by motion compared to the Cartesian sampling patterns employed in this study (40). Alternatively, acceleration can be utilized to enhance spatial resolution, which is especially relevant for T1 mapping of the right ventricle (RV) or the atria, aiming to detect fibrosis (41). Although challenging due to the thin RV wall, this improvement in spatial resolution can hold great significance in the evaluation of conditions such as pulmonary artery hypertension or arrhythmogenic right ventricular dysplasia.

## Limitations

The analysis conducted in this study had certain limitations. Firstly, the number of patients with HCM was limited, which hindered a comprehensive quantitative analysis of relaxation time values. To obtain a more comprehensive understanding, future studies should aim to include patients with diverse myocardial conditions, varying heart rates, presence or absence of arrhythmias, and a wide range of body weights. This is specifically important because our healthy subjects had a BMI of approximately 22, whereas larger FOVs needed for patients with higher BMIs will have a negative impact on the performance of PI. This would allow for the exploration of accuracy and precision limits across a heterogeneous patient population.

Furthermore, due to the current study protocol, it was not possible to establish the repeatability of accelerated scans among study participants. However, we designed the study protocol based on favorable agreement observed in phantom measurements and took measures to minimize scan duration to prioritize the well-being of the study participants. Additionally, it is important to note that restrictions on the repeated administration of gadolinium in cardiac patients will make it more challenging to perform test-retest accelerated scans.

## Conclusion

By employing our newly developed 72-channel receiver array coil in conjunction with SENSE acceleration up to a factor of 6, we were able to achieve time-efficient tissue characterization of the left ventricle myocardium across three slices. This advanced setup allowed us to acquire pre- and post-contrast T1 maps, each within a single BH, facilitating ECV calculation. T2 maps could be acquired in two consecutive BHs. This approach demonstrates the feasibility of acquiring multiple quantitative maps efficiently and with reduced patient burden.

## Data Availability

The raw data supporting the conclusions of this article will be made available by the authors, without undue reservation.
